# Selective human milk oligosaccharide utilization by members of the *Bifidobacterium pseudocatenulatum* taxon

**DOI:** 10.1128/aem.00648-24

**Published:** 2024-09-24

**Authors:** Rocio Sanchez-Gallardo, Francesca Bottacini, Ian J. O’Neill, Maria Esteban-Torres, Rebecca Moore, Fionnuala M. McAuliffe, Paul D. Cotter, Douwe van Sinderen

**Affiliations:** 1 APC Microbiome Ireland, University College Cork, Cork, Ireland; 2 School of Microbiology, University College Cork, Cork, Ireland; 3 Biological Sciences and ADAPT, Munster Technological University, Cork, Ireland; 4 UCD Perinatal Research Centre, School of Medicine, University College Dublin, National Maternity Hospital, Dublin, Ireland; 5 Teagasc Food Research Centre Moorepark, Cork, Ireland; The Pennsylvania State University, University Park, Pennsylvania, USA

**Keywords:** bifidobacteria, human milk oligosaccharides, early life gut colonization, gut microbiota

## Abstract

Human milk oligosaccharides (HMOs) are essentially unaffected by the digestive enzymes of the nursling and are known for their ability to enrich certain microbial species in the infant gut microbiota, in particular bifidobacteria. HMO metabolism has been studied in various bifidobacterial species such as *B. breve*, *B. bifidum,* and *B. longum* subsp. *infantis*. In the current study, we describe differential growth abilities elicited by twenty-three newly isolated *Bifidobacterium pseudocatenulatum* strains on particular HMOs, such as 2’-fucosyllactose (2’FL), 3-fucosyllactose (3FL), lacto-N-tetraose (LNT), and lacto-N-neotetraose (LNnT). Through gene-trait matching and comparative genome analysis, we identified genes involved in the degradation of fucosylated HMOs in this strain set, while we employed a transcriptomic approach to facilitate the identification and characterization of genes and associated enzymes involved in LNT metabolism by strain *B. pseudocatenulatum* MM0196. A total of 252 publicly available genomes of the *B. pseudocatenulatum* taxon were screened for homologs of the glycosyl hydrolases (GHs) identified here as being required for selected HMO metabolism. From this analysis, it is clear that all members of this species possess homologs of the genes involved in LNT degradation, while genes required for degradation of fucosylated HMOs are variably present.

**IMPORTANCE:**

Our findings allow a better understanding of the complex interaction between *Bifidobacterium* and its host and provide a roadmap toward future applications of *B. pseudocatenulatum* as a probiotic with a focus on infant health. Furthermore, our investigations have generated information on the role of HMOs in shaping the infant gut microbiota, thus also facilitating applications of HMOs in infant nutrition, with potential extension into the mature or adult gut microbiota. Supplementation of HMOs is known to result in the modulation of bacterial communities toward a higher relative abundance of bifidobacteria, which in turn enforces their ability to modulate particular immune functions and strengthen the intestinal barrier. This work may therefore inspire future studies to improve the formulation of neonatal nutritional products, aimed at facilitating the development of a healthy digestive and immune system and reducing the differences in gut microbiota composition observed between breastfed and formula-fed babies or full-term and preterm infants.

## INTRODUCTION

Human milk oligosaccharides (HMOs) constitute a large group of structurally complex glycans that are exclusively present in human breast milk. Each lactating woman produces a unique combination of HMOs that vary in composition and quantity. Various factors such as lactation stage, diet of the mother, or genetic aspects influence the specific amount and precise composition of HMOs produced by each lactating mother (1). These carbohydrates are the second most abundant sugars found in human milk after lactose, and they are considered pivotal in promoting the establishment of a healthy infant gut microbiota.

HMOs are resistant to digestive enzymes produced by the infant and are only absorbed by the host in small quantities, thus essentially reaching the colon intact where particular elements of the microbiota are able to metabolize (some of) these complex oligosaccharides. HMOs and the microbial elements they support are recognized to exert a range of benefits upon the developing neonate, for example, production of lactic acid and short-chain fatty acids, and modulation of neonatal immunity by altering host epithelial and immune cell responses. Disruptions of the latter processes may have a negative impact on later life health, such as allergy development or impaired cognitive function (2, 3).

Over two hundred distinct HMO structures have been described to date (4). This structural diversity among HMOs is due to size, glycosidic linkages, and varying substitutions and monosaccharide building blocks, the latter typically represented by five basic units: sialic acid, N-acetylglucosamine, L-fucose, D-galactose, and D-glucose. All HMOs contain a lactose core at the reducing end, and this core can be extended by lacto-N-biose or N-acetyllactosamine and/or substituted by fucose and/or sialic acid. HMOs containing sialic acid moieties are considered sialylated oligosaccharides, rendering them acidic, while those that harbor a fucose molecule are termed fucosylated HMOs (FHMOs) and together with non-fucosylated and non-sialylated HMOs represent neutral oligosaccharides (4, 5).

The feeding mode, i.e., breast or formula feeding, is one of the main factors influencing the composition of the infant microbiota. Breastfed infants tend to have a higher prevalence and relative abundance of bifidobacteria and a different metabolome compared to formula-fed infants (6). Bifidobacteria are among the first colonizers of the neonatal gut, in part due to their ability to digest HMOs. Members of the species *Bifidobacterium breve*, *Bifidobacterium bifidum,* and *Bifidobacterium longum* (subspecies *longum* and *infantis*) are commonly isolated from infant fecal samples and are among the most abundant species found in early life gut microbiota. As HMO degradation is believed to play a key role in gut colonization by and persistence of *Bifidobacterium,* the metabolic pathways underlying this ability have been the subject of intense study (7). Various HMO degradative pathways exist among different bifidobacterial species. Bifidobacterial HMO metabolism may exclusively take place intracellularly: this involves specific ABC-type transporters to internalize particular HMOs, which are then hydrolyzed by dedicated glycosyl hydrolases (GHs) into individual monosaccharides. Most of these carbohydrates (all except fucose) are then either directly or indirectly incorporated into the Bifid shunt, which represents the central and unique pathway for carbohydrate metabolism employed by bifidobacteria. In contrast, certain bifidobacteria, such as *B. bifidum*, employ extracellular GHs that act upon HMOs outside of the cell to mainly release mono- and di-saccharides, which are then transported into the cytoplasm (mostly) by dedicated ABC transporters before entering the Bifid shunt. This latter mechanism of HMO degradation allows other (bifido)bacteria to cross-feed on such extracellularly released carbohydrates and coexist despite lacking HMO-degrading abilities (8, 9).

Following weaning (i.e., the cessation of a milk-only diet by the incorporation of solid foods), the infant gut microbiota changes, which also affects bifidobacterial abundance and species composition (10). Infant-associated species become less abundant, and other adult-associated (bifido)bacterial species such as *Bifidobacterium adolescentis* become more prominent as they are capable of digesting dietary plant-derived carbohydrates. Some studies have indicated that *Bifidobacterium pseudocatenulatum* is a bifidobacterial species whose prevalence and relative abundance remain relatively constant across the lifetime of a person (11). Thus, it is expected that this species possesses an extensive enzymatic machinery to adapt to dietary changes. Some *B. pseudocatenulatum* strains have been reported to metabolize certain HMOs such as 2’-fucosyllactose (2′-FL), 3-fucosyllactose (3-FL), and lacto-N-tetraose (LNT).(12) *B. pseudocatenulatum* DSM20438 is a strain that can metabolize 2′-FL and 3-FL, a phenotype which has been linked to the presence of a particular FHMO utilization gene cluster, similar to the one described for *B. longum* subsp. *infantis*, and typified by the presence of a gene encoding an α-fucosidase belonging to the GH95 family (13). A recent study identified homologs of this cluster in *B. pseudocatenulatum* strains, with the peculiarity that in one case, the cluster was shown to encode a predicted GH29 family α-fucosidase, similar to the one present in *B. kashiwanohense* APCKJ (12, 14). The presence of this predicted GH29 fucosidase-encoding gene was speculated to endow this strain with an ability to consume a larger repertoire of fucosylated structures within a pool of HMOs (12). While LNT catabolism has previously been studied in other bifidobacterial species such as *B. breve* UCC2003, the precise mechanism or genes involved in the degradation of this HMO is still unknown in *B. pseudocatenulatum* (15).

The current study provides information on HMO utilization abilities of a set of *B. pseudocatenulatum* strains, previously obtained from the so-called Microbe Mom trial (16–18), by uncovering genes and encoded GHs involved in their degradative pathways.

## RESULTS

### Growth of *B. pseudocatenulatum* strains on different HMOs

**Fig 1:**
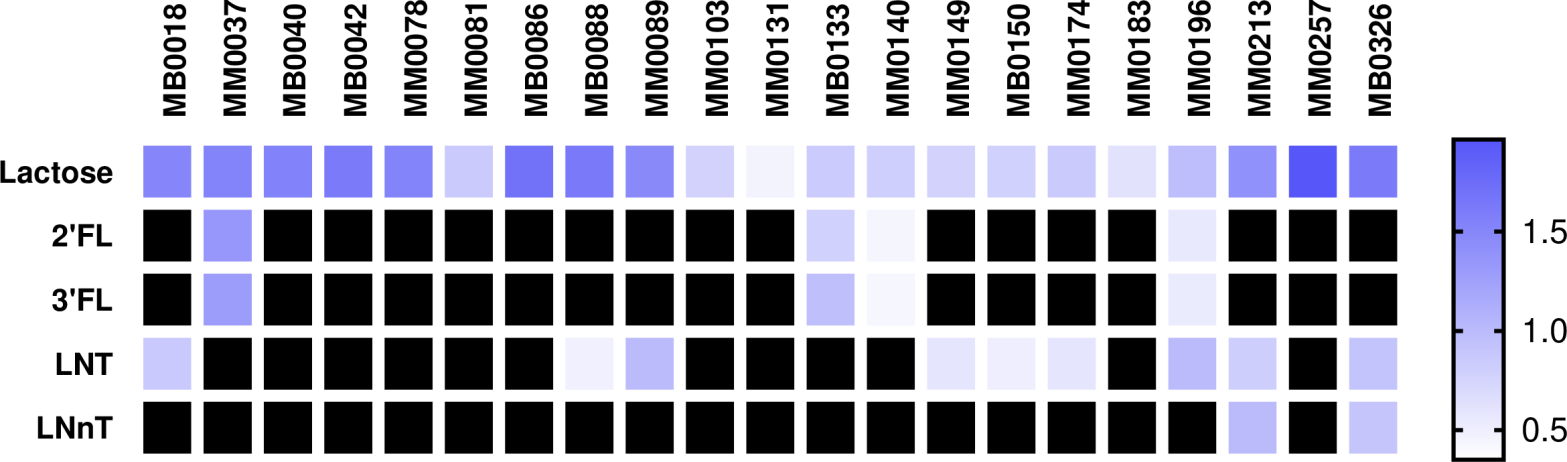
Heatmap showing the growth performance of 21 *B. pseudocatenulatum* strains on different HMOs used as carbon sources at 24 hours. Blue color represents growth according to the OD indicated in the legend, and black indicates no growth (cutoff of 0.35 OD) This heatmap represents the average OD obtained of three independent experiments.

Previously, 21 distinct strains of *B. pseudocatenulatum* had been isolated from a number of mother–infant dyads (16–18). In order to investigate the HMO-metabolizing abilities of these *B. pseudocatenulatum* isolates, they were assessed for growth in modified MRS medium (mMRS) supplemented with 0.5% (wt/vol) of a given HMO as the sole carbon source. The HMOs used were 2’FL, 3FL, LNT, and LNnT, with lactose and no carbohydrate being employed as positive and negative controls, respectively. Growth was assessed by measuring the optical density at 600 nm after 24 hours of anerobic incubation at 37 degrees Celsius. The tested strains were shown to exhibit varying HMO utilization patterns, as displayed in Fig. 1. Only four out of the 21 tested *B. pseudocatenulatum* strains, i.e., MM0037, MM0140, MM0133, and MM0196, were capable of growth in 2’FL and 3FL. Nine were able to grow in LNT, while only two strains exhibited growth in LNnT. Under the conditions tested, strain MM0196 was shown to be the only strain able to use 2’FL, 3FL, and LNT.

### Growth and genetic organization of the FHMO cluster in *B. pseudocatenulatum*

Two clusters have been described to date in *B. pseudocatenulatum* genomes responsible for FHMO utilization. The first cluster is identical to the FHMO utilization cluster described for *B. catenulatum* subsp. *kashiwanohense* APCKJ1 and encodes two α-fucosidases (GH29 and GH95), while the second FHMO cluster is that identified in *B. pseudocatenulatum* DSM20438, which lacks two genes compared to the first cluster, encoding an α-fucosidase belonging to the GH family 29 and a predicted L-fucose mutorotase (12, 14).

**Fig 2:**
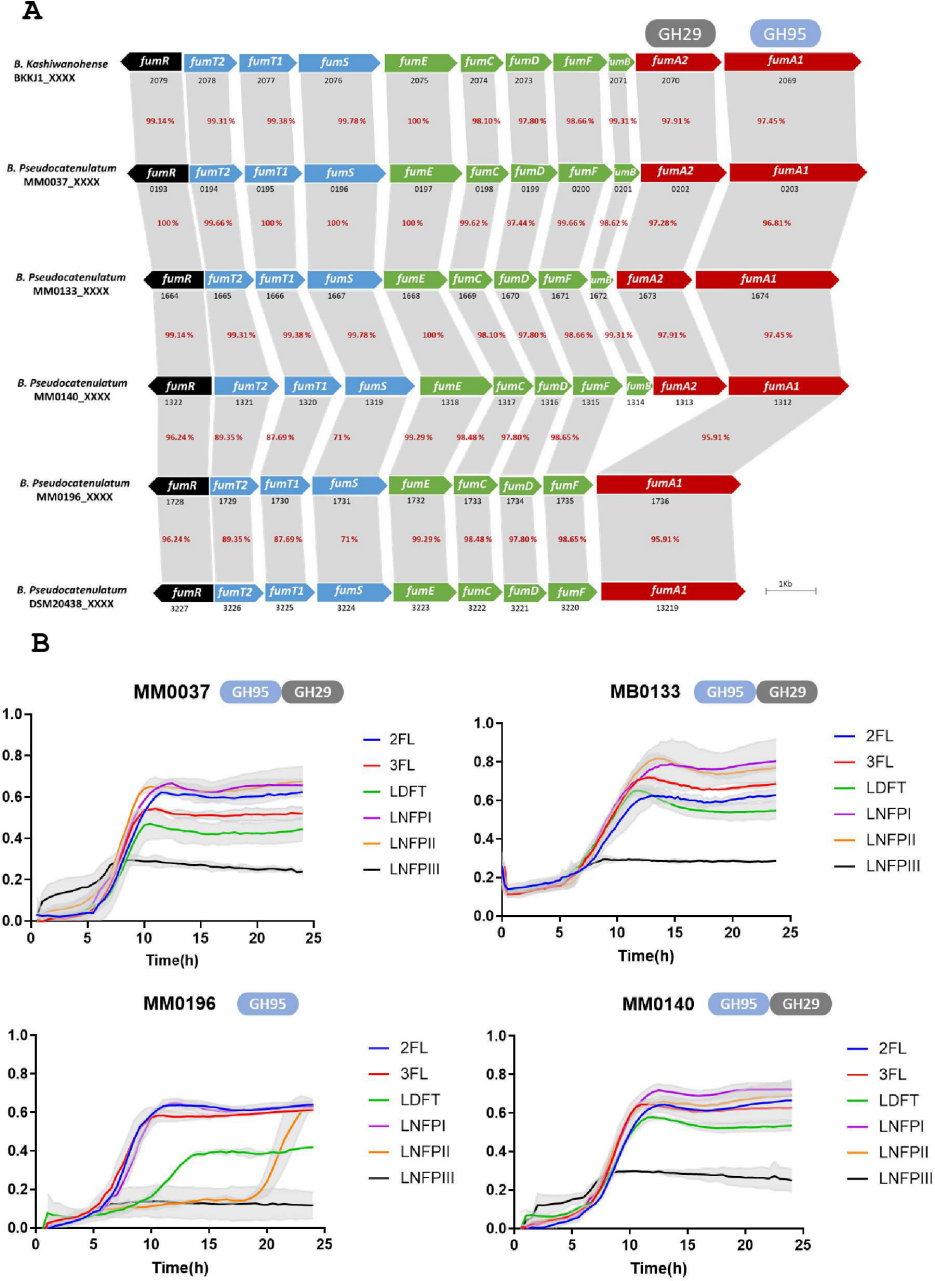
(A) Genetic representation of the FHMO utilization cluster in *B. kashiwanohense* APCKJ1 and homologous genes in *B. pseudocatenulatum* strains isolated in this study. Arrows represent the genes, and numbers on top of each gene indicate the locus tag number on the respective genome. The color of each gene is indicative of their function: transcriptional regulators (black), oligosaccharide transport (blue), carbohydrate feeder pathways (green), and glycosyl hydrolases (red). The percentage of identity corresponds with the identity with the cluster from *B. catenulatum* subsp. *kashiwanohense* APCKJ1. (B) Growth profile of *B. pseudocatenulatum* strains that have a FHMO cluster in their genome. Growth was assessed in new modified Rogosa medium supplemented with 0.5% (wt/vol) of each of the carbohydrates specified on the legend. The graph shows the average of three independent experiments.

The functionality of the FHMO cluster of *B. catenulatum* subsp. *kashiwanohense* APCKJ1 has previously been investigated (14). In summary, 2’FL and 3FL are incorporated intracellularly through a specific transporter. The fucose molecule is then removed from 2’FL/3FL by one of the two fucosidases (*fumA1/fumA2*), releasing lactose. The liberated fucose is transformed into L-fuconolactone by FumB and FumC, which is then modified by FumD into L-fuconate. FumE is responsible for conversion of L-fuconate into L-2-keto-3-deoxy-fuconate, which is then transformed into L-lactaldehyde and pyruvate by FumF (Fig. 2A). FumG would later reduce L-lactaldehyde into L-1,2-propanediol, despite not being present in the cluster, and previous studies confirm the key role of this protein in the metabolism of FHMO (14).

As described above (Fig. 1), only four out of twenty-one *B. pseudocatenulatum* strains included in this study were shown to metabolize 2’FL and 3FL, so their genomes were scrutinized for the presence of genes encoding GH29/GH95 members. All four strains were found to possess a gene encoding a GH95 in their genome, while in the cases of MM0037, MM0133, and MM0140, an additional gene was present that was predicted to encode a second fucosidase belonging to the GH family 29, being located directly upstream of the gene encoding the GH95 enzyme. To assess the degree of similarity and synteny between these two putative FHMO gene clusters of *B. pseudocatenulatum* and those described for the species *B. catenulatum* subsp. *kashiwanohense*, we performed a BLASTP-mediated comparative sequence analysis (Fig. 2A) (13). The percentage of identity is above 95 percent in all encoded products for strains MM0037, MM0133, and MM0149; however, strain MM196 lacks the GH29-encoding gene as well as a small gene encoding a putative L-fucose mutanorotase, while the identity percentage of the predicted ABC-type transporters is lower, ranging from 71% in the case of *fumS* to 87% and 89% in the case of *fumT2* and *fumT1*, respectively.

Interestingly, the cluster present in MM0196 is identical to the one present in DSM20438, while the cluster present in the strains MM0037, MM0133 ,and MM0149 is identical to the one previously characterized in *B. catenulatum* subsp. *kashiwanohense* APCKJ1 (14).

To further investigate if the predicted production of a second fucosidase by certain *B. pseudocatenulatum* strains allows the metabolism of a wider range of FHMOs, strains MM0037, MM0133, MM0149, and MM0196 were assessed for growth in a medium containing other FHMOs, in particular difucosyllactose (LDFT), lacto-N-fucopentaose I (LNFPI), lacto-N-fucopentaose II (LNFPII), and lacto-N-fucopentaose III (LNFPIII). In this case, the optical density as a measure of growth was determined using a plate reader (see Materials and Methods). The main difference between the FHMO-supported growth patterns obtained for these strain concerns growth on LNFPII. This HMO elicits the same growth profile by all strains, except for MM0196. In the latter case, a longer lag phase was observed, with growth initiating after 20 hours of incubation (Fig. 2B). Furthermore, despite the fact that all strains were shown to utilize DFL, strain MM0196 reached an OD600 nm of just 0.4 and exhibited a longer lag phase, when compared to the growth characteristics elicited by the other four assessed strains. None of the strains were able to utilize LNFPII.

The observed differential growth characteristics among strains that encode different fucosidases suggest that the GH29 fucosidase plays a role in LNFPII utilization, while also affecting the ability to grow in DFL. This indicates that the presence of both fucosidase-encoding genes*, fumA1* and *fumA2*; the L-fucose mutanorotase, and probably the specificity of the transporter present in the cluster allows the strain to utilize a wider range of FHMOs.

### Transcriptome analysis of *B. pseudocatenulatum* MM0196 in response to LNT supplementation

**Fig 3:**
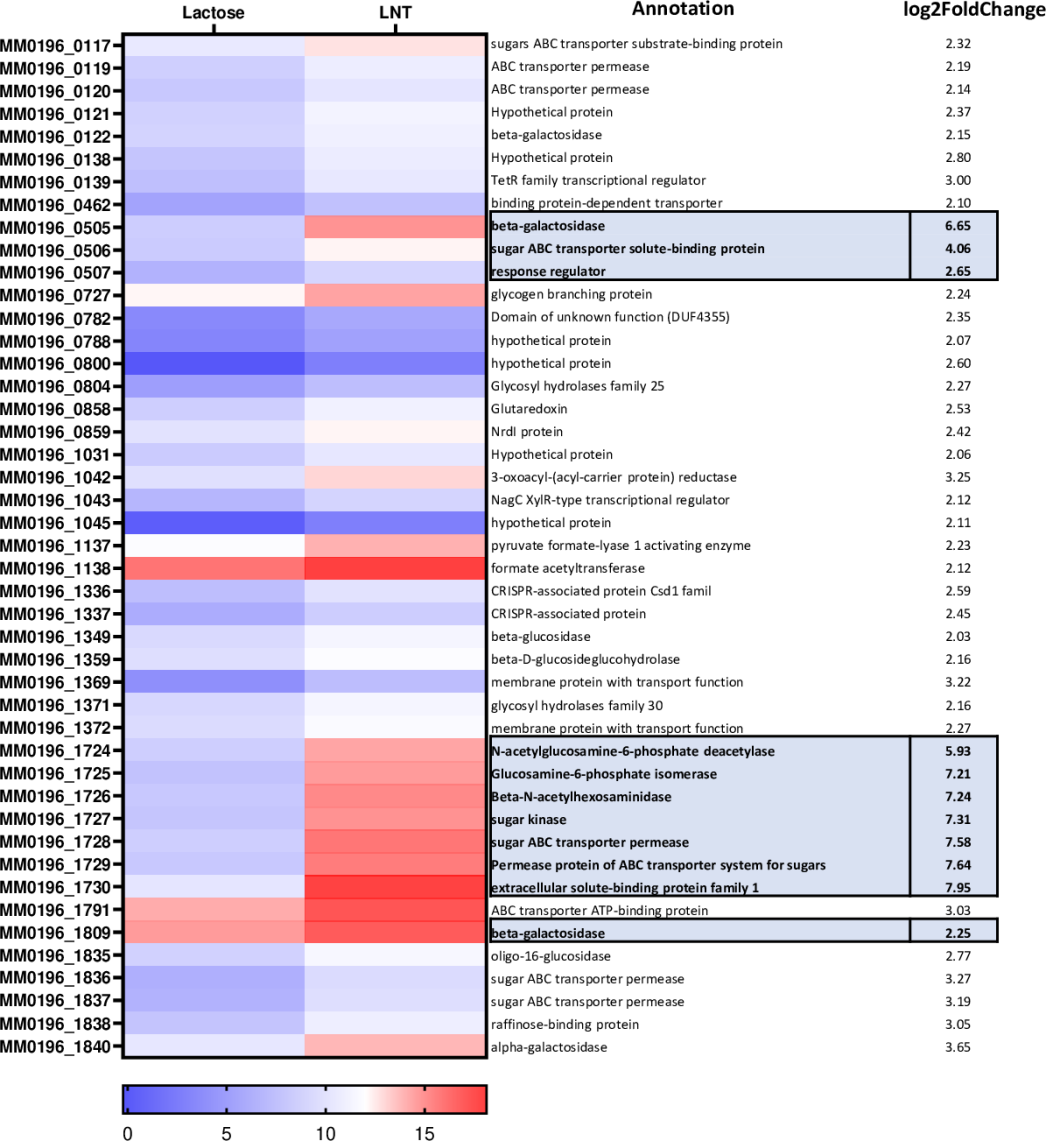
Heatmap representing the logarithm in base 2 of the reads obtained as a result of the RNA-seq. *B. pseudocateulatum* MM0196 was grown in LNT and lactose. Color scale specified on the legend. Blue represents low expression level and red higher. On the right column, the level of expression is shown as a fold-value of an increase in the expression on LNT to a lactose control, with a cut-off of a minimum twofold increase in expression. GHs within the three loci focused on in this study are shown in bold script (complete data set in the supplementary material).

An initial *in silico* analysis based on sequence homology with genes known to be involved in LNT metabolism in other *Bifidobacterium* species allowed the identification of potential candidates involved in the metabolism of this HMO; however, due to the lack of any obvious gene–phenotype matches, we were unable to identify the genes responsible for this trait. Therefore, transcriptomic analysis was undertaken using RNA-seq to identify genes whose transcription was upregulated in the presence of LNT in strain MM0196 compared to the transcriptome obtained for this strain when grown on lactose. The resulting differential expression analysis revealed a total of 121 differentially expressed genes (DEGs), consisting of 45 upregulated and 76 downregulated genes (Fig. 3A). Several of the upregulated genes were clustered into three genomic regions. In line with previous studies, three of the upregulated genes were predicted to encode GHs, the function of which could be linked to LNT metabolism (15). These were (genes with locus tag number) MM0196_0505, which encodes a predicted GH42 familyβ-galactosidase; MM0196_1726, specifying a predicted GH20 N-acetylglucosaminidase; and MM0196_1809, encoding a predicted GH2 β-galactosidase. MM0196_0505 is located in this first genomic region and is adjacent to genes that encode an ABC transporter (MM0196_0506) and a response regulator (MM0196_0507). The second genomic region harbors a total of seven upregulated genes predicted to encode an N-acetylglucosamine-6-phosphate deacetylase (MM0196_1724), a glucosamine-6-phosphate isomerase (MM0196_1725), a GH20 β-N-acetyl hexosaminidase (MM0196_1726), a sugar kinase (MM0196_1727), two ABC transporter permease (MM0196_1728 and MM0196_1729), and a solute-binding protein (MM0196_1730). In the third region, a beta-galactosidase (MM0196_1809) responsible for the cleavage of lactose can be found.

In order to assess if the presence of these genes is associated with the obtained phenotypic data described above, as some of the strains were not able to utilize LNT, a genomic comparative analysis was performed to locate the orthologous genes across the *B. pseudocatenulatum* genomes employed in this study. Somewhat to our surprise, all assessed genes are present in all scrutinized *B. pseudocatenulatum* genomes, except for the gene with locus tag MM0196_0507, a predicted regulator that is absent in MB0018, MB0150, MB0326, MM0131, MM0149, MM0174, and MM0327. These findings therefore show that there is no apparent correlation between the presence or absence of LNT-upregulated genes and the ability of that strain to grow in LNT (Fig. S1).

A comparative analysis reveals that the genes encoding LNT-associated GHs identified in *B. pseudocatenulatum* MM0196 exhibit similarity to those previously described in *B. breve* UCC2003 as being responsible for LNT metabolism (Table 1). These were *lntA*, the most similar to the orthologs in the strain MM0196 with 76.38% identity, followed by *nahA*, with 60% identity, and *lacZ6* exhibiting 54.67% identity. Based on the observed sequence similarities, we used the same names for corresponding orthologous genes present in *B. pseudocatenulatum* MM0196 (though using the subscript “ps” to distinguish then, e.g., *lntA_ps_*). Despite the high resemblance of these GH-encoding genes, some differences can be observed regarding their genetic organization and the similarity of certain associated genes, such as transcriptional regulators. Three clusters/genomic regions were identified in the genome of *B. breve* UCC2003, while in strain MM0196, the orthologous genes are distributed differently in the genome.

**TABLE 1:** Comparison of transcriptionally upregulated *B. breve* UCC2003 genes during growth in mMRS medium supplemented with 1% LNT with the genes that are also upregulated in *B. pseudocatenulatum* MM0196 when grown on LNT, indicated in bold

Annotation	Gene name	UCC2003	MM0196	Percentage identity
Transcriptional regulator, LacI family	*lntR*	Bbr_0526	MM0196_1515	29.06
Permease protein of ABC transporter system for sugars	*lntP1*	Bbr_0527	MM0196_1518	88.27
Permease protein of ABC transporter system for sugars	*lntP2*	Bbr_0528	MM0196_1517	86.15
**GH42 Beta-galactosidase**	*lntA*	Bbr_0529	**MM0196_0505**	**76.38**
Solute-binding protein of ABC transporter system for sugars	*lntS*	Bbr_0530	MM0196_0506	51.80
Galactoside symporter	*lacS*	Bbr_1551	MM0196_1808	66.95
**GH2 Beta-galactosidase**	*LacZ6*	Bbr_1552	**MM0196_1809**	**54.67**
Transcriptional regulator, LacI family	*lacI*	Bbr_1553	MM0196_1810	47.65
Solute-binding protein of ABC transporter system (lactose)	*nahS*	Bbr_1554	MM0196_1519	38.80
NagC/XylR-type transciptional regulator	*nahR*	Bbr_1555	MM0196_1731	46.65
**GH20 nagZ Beta-N-acetylhexosaminidase**	*nahA*	Bbr_1556	**MM0196_1726**	**60.75**
Phosphotransferase family protein	*nahK*	Bbr_1586	MM0196_1440	22.55
GH112 lacto-N-biose phorylase	*lnbP*	Bbr_1587	MM0196_0465	27.12
Permease protein of the ABC transporter system for sugars	*galP1*	Bbr_1588	MM0196_1728	87.37
Permease protein of the ABC transporter system for sugars	*galP2*	Bbr_1589	MM0196_1729	87.46
Solute-binding protein of the ABC transporter system for sugars	*galS*	Bbr_1590	MM0196_1730	70.18

### Heterologous expression, purification, and biochemical characterization of the gene products of *lntA_ps_* (MM0196_0505), *nahA_ps_* (MM0196_1726), and *lacZ6_ps_* (MM0196_1809)

Transcriptomic results facilitated the identification of the glycosyl hydrolases potentially involved in the degradation of LNT. The gene products of *lntA_ps_* (MM0196_0505), *nahA_ps_* (MM0196_1726), and *lacZ6_ps_* (MM0196_1809) were heterologously expressed in *Lactococcus cremoris* NZ9000 in order to investigate their enzymatic activity and confirm their predicted function. Following purification, the resulting His-tagged proteins were used in enzymatic reactions using LNT or lactose as substrates. The products of these enzymatic reactions were then analyzed using HPAEC-PAD.

**Fig 4:**
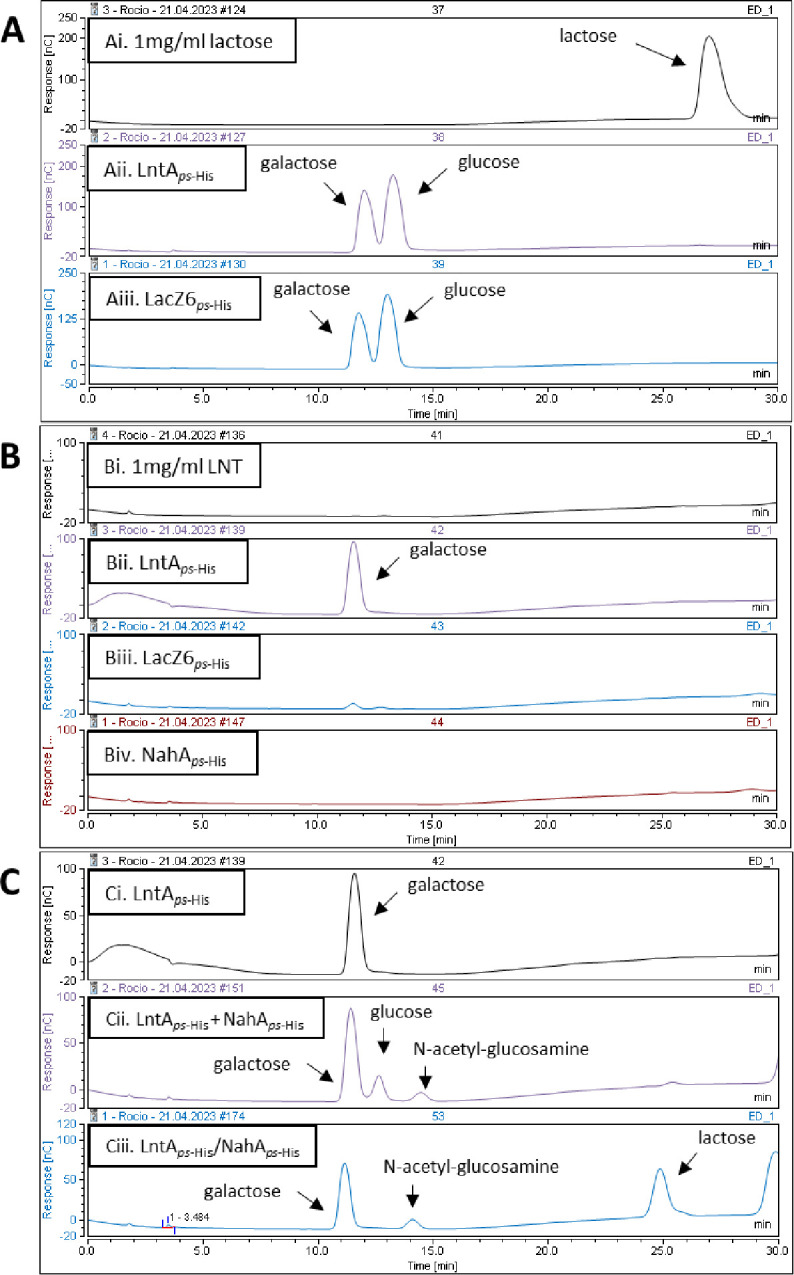
HPAEC-PAD chromatogram profiles obtained after 24-hour incubation of a given carbohydrate with relevant enzyme at 37°C. (A) (Ai) Lactose (control); lactose-derived products following incubation with (Aii) LntA_ps-His_ or (Aiii) LacZ6_ps-His_. (B) (Bi) LNT (control); LNT-derived products following incubation with (Bii) LntA_ps-His_, (Biii) LacZ6_ps-His_, or (Biv) NahA_ps-His_. (C) LNT-derived products following incubation with (Ci) LntA_ps-HHis_, (Cii) combination of LntA_ps-His_ and NahA_ps-His_, or (Ciii) LntA_ps-His_ incubated for 24 hours; after deactivation of the first enzyme, NahA_ps-His_ was added, followed by another 24 hours of incubation.

Purified LntA*_ps-_*_His_ and LacZ9A*_ps-_*_His_ were shown to cleave lactose, releasing glucose and galactose, confirming their predicted activity as a β-galactosidase (Fig. 4A). When each of these enzymes was incubated with LNT, only LntA*_ps-_*_His_ was shown to be able to remove the galactose moiety at the non-reducing end of the LNT substrate (Fig. 4B), demonstrating that this gene product has hydrolytic activity not only toward lactose but also toward LNT.

No degradation was observed by HPAEC when NahA*_ps-_*_His_ was incubated with LNT (Fig. 4Biv). However, when this enzyme was incubated together with LntA*_ps-_*_His_ in the presence of the LNT substrate, N-acetylglucosamine, galactose, and glucose were produced (Fig. 4Cii). This result confirms the predicted glycolytic activity of NahA*_ps-_*_His_ as an N-acetylglucosaminidase, which removes N-acetylglucosamine from LNT, yet only if the galactose at the non-reducing end of LNT has been removed first by LntA*_ps-_*_His_, releasing lactose, which is then cleaved by LntA*_ps-_*_His_ into galactose and glucose.

To validate the above-indicated sequential LNT hydrolysis steps, we first incubated LntA*_ps-_*_His_ with LNT, then subjected the reaction to a denaturation step (85°C for 15 minutes) to inactivate LntA*_ps-_*_His_, after which NahA*_ps-_*_His_ was added and incubated under the appropriate reaction conditions. This experimental approach resulted in LNT being degraded into galactose, N-acetylglucosamine, and lactose (Fig. 4Ciii). In this case, NahA*_ps-_*_His_ can remove N-acetylglucosamine from lacto-N-triose produced by LntA*_ps-_*_His_, releasing lactose, yet the latter disaccharide is now not further hydrolyzed by LntA*_ps-_*_His_ as this enzyme is no longer active following the heating step.

### Distribution of genes encoding GHs involved in HMO degradation across all publicly available *B. pseudocatenulatum* genomes

As described above, we identified *lntA_ps_*, encoding an LNT-specific β-galactosidase, and *nahA_ps,_* encoding an N-acetylhexosaminidase, as key genes involved in the catabolism of LNT by *B. pseudocatenulatum* strain MM0196. Furthermore, the two main GHs involved in the degradation of FHMOs, encoded by *fumA1_ps_* and *fumA2_ps_*, were reported to be present in some of the assessed *B. pseudocatenulatum* strains.

**Fig 5:**
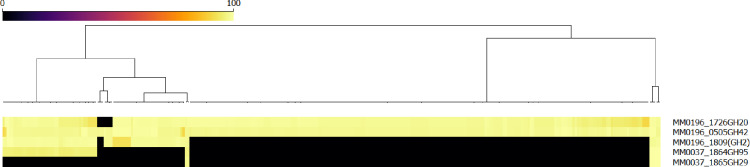
Presence (yellow) or absence (black) of glucosyl hydrolases involved in the HMO metabolism in all publicly available genomes of *B. pseudocatenulatum*. See also Table S1.

In order to determine how prevalent the above-mentioned GH-encoding genes for LNT and FHMO metabolism are found in members of the *B. pseudocatenulatum* taxon, their presence was investigated in all publicly available *B. pseudocatenulatum* genomes (252 genomes), through a blastp-based analysis (see *Materials and Methods*). The results obtained with a percentage of identity higher than 80% and a coverage over 90% are represented in a heatmap (Fig. 5).

From the obtained results, it is obvious that *lntA* is fully conserved among all strains assessed. The *nahA* gene was also shown to be highly conserved, apparently absent in three strains out of the 252, although this may also be due to the incomplete nature of some of the examined genomes. This indicates that there may have been other factors that influence their ability to degrade LNT and thus the ability to grow in LNT under the conditions tested here.

In contrast, in the two genes involved in FHMO degradation (as representatives of the cluster), we observed that neither is highly conserved among the *B. pseudocatenulatum* species. Interestingly, *fumA1_ps_* was shown to be more prevalent compared to *fumA2_ps_*, the former being present in forty-seven strains of 252 included in this analysis, representing 18% of the publicly available genomes. The *fumA2_ps_* gene was shown to be present in just five out of 252 examined strains, representing 2% of the total *B. pseudocatenulatum* genomes (of which two correspond to publicly available genomes, with the remaining three being the strains MM0037, MM0133, and MM0140, described herein).

## DISCUSSION

HMO consumption by gut commensals is believed to be one of the key factors contributing to the development of a healthy infant gut, in particular through modulation of bacterial communities toward a bifidobacteria-rich microbiota. The latter are represented by strains belonging to typically, infant-associated species such as *B. breve*, *B. longum* subsp. *infantis,* and *B. bifidum*, which are associated with health-promoting attributes, especially during early life (10, 19).

Our work shows that members of the *B. pseudocatenulatum* taxon can also degrade certain HMO structures. *B. pseudocatenulatum* is considered an adult-associated bifidobacterial species, as it is frequently isolated from or detected in the adult gut/feces, despite the fact that many strains have been reported to be present in infant feces (10, 12). This is most likely due to their conserved ability to degrade plant-derived carbohydrates, which seems to be a common trait across this bifidobacterial species (20–23). This view is partly supported by our observations and is consistent with findings reported by others and shows that their ability to degrade HMOs is strain-dependent and in general appears to be a rather rare trait across this species (12).

As described here, all examined members of this species possess the genes responsible for LNT degradation. However, this genotype was shown not to be reflected in the observed phenotype of strains when their growth was assessed under the conditions described above. This suggests that expression of these genes may require induction by an as yet unknown environmental signal. Despite the conservation observed across bifidobacterial species with respect to the sequence and structure of the FHMO cluster (12, 13), we describe that two variations of the cluster, mostly connected with the presence/absence of a second α-fucosidase (GH29), are associated with the metabolism of an expanded set of FHMO structures. A previous study on the specificity of the two GHs showed that the α-fucosidase (GH95) encoded by the corresponding gene cluster exhibits a lower substrate affinity for LNFII, likely explaining the longer lag phase observed for the strains lacking this gene (12). While all tested strains were able to grow in LNFPI, it is interesting to note that none of the strains were shown to be capable of utilizing LNFPIII, suggesting that a different transporter is required, as the same fucose substitution can be found in 3FL or may be due to the substrate specificity/affinity with this particular structure. To date, growth of *B. pseudocatenulatum* on this HMO has not been reported.

**Fig 6:**
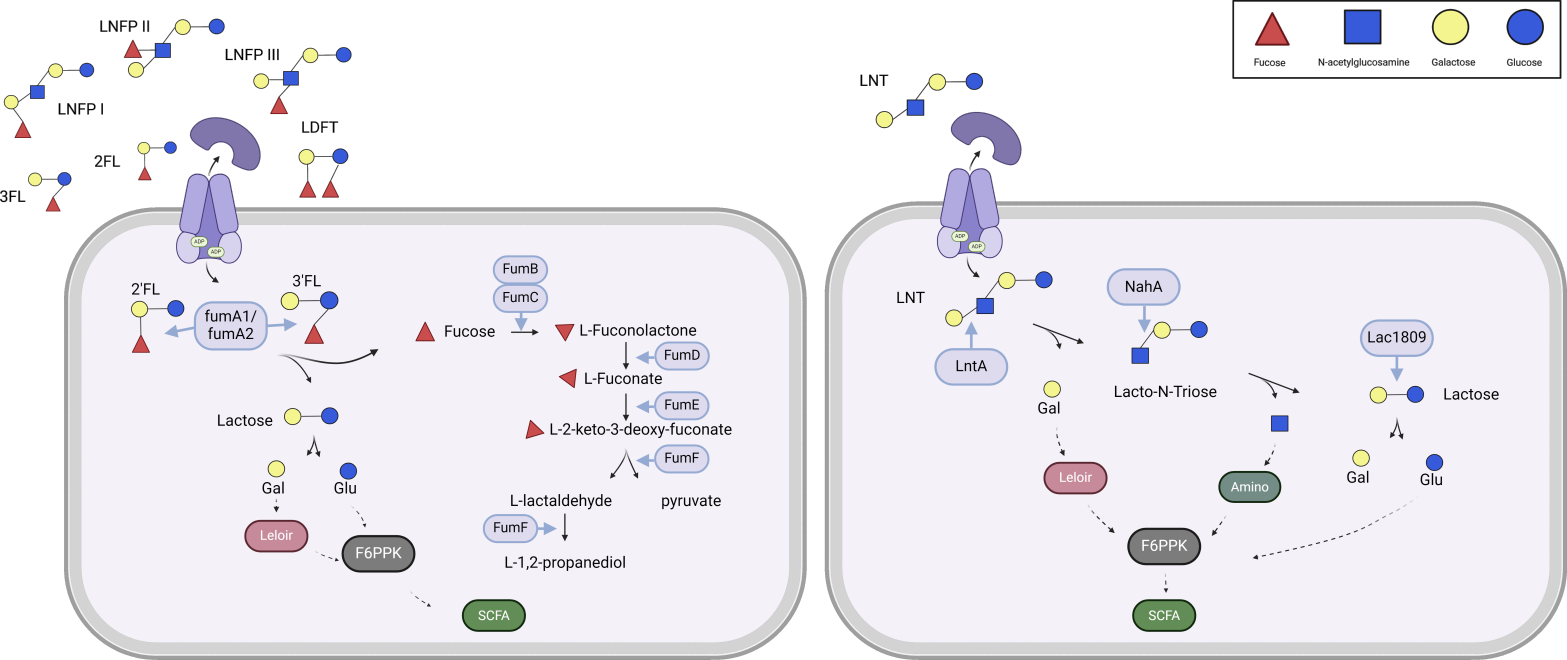
Representation of the HMO metabolism in the *B. pseudocatenulatum* (Left) degradation pathway described in *B. kashiwanohense* homologous in *B. pseudocatenulatum* (14). (Right) Utilization pathway in *B. pseudocatenulatum,* similar to the one elicited by *B. breve* (15).

Through RNA-seq analysis, we were able to identify genes involved in LNT degradation by the commensal *B. pseudocatenulatum* MM0196. Transcription of *lntA_ps_*, *nahA_ps,_* and *lacZ6_ps_* was shown to be upregulated when this strain was grown in LNT, and the predicted function of their encoded products as specific GHs was then verified by heterologous expression, protein purification, and enzymatic reaction using LNT as the substrate. This analysis led to the conclusion that the predicted lntA_ps-His_ b-galactosidase cleaves galactose from the LNT molecule, releasing lacto-N-tetraose, and that NahA_ps-His_, a predicted N-acetyl hexoaminidase, is then able to remove N-acetylglucosamine from lacto-N-triose, releasing lactose. Both enzymes used in combination and subsequently released the expected product. When purified, the third upregulated GH lacZ9_ps-His_ was shown to hydrolyze lactose into glucose and galactose, which is then the last step of degradation of this HMO before incorporation into the bifid shunt to obtain energy (Fig. 6, right). This LNT degradation pathway is similar to that previously described for *B. breve* UCC2003 (15).

The presence of highly homologous genes to those described in MM0196 in other *B. pseudocatenulatum* strains does not always correspond with their ability to grow in LNT, as observed in the current experimental conditions. This finding suggests that the expression of these genes might be influenced or triggered by environmental factors.

When the comparative genomic analysis was extended to all publicly available genomes from the species *B. pseudocatenulatum*, we observed a similar pattern, where homologs of the GHs involved in the LNT degradation were identified in (nearly) every single genome. Although we could not phenotypically assess all of these strains, their conserved nature suggests that these genes have a fundamental role in this bifidobacterial species and implies that, in principle, most if not all *B. pseudocatenulatum* strains are able to degrade LNT.

In contrast, the conservation of the GH-encoding gene present in the FHMO cluster is very low among the *B. pseudocatenulatum* genomes, with the predicted GH95-encoding gene found to be present in forty-seven publicly available strains (out of 252), and four of this study, while the GH29-encoding gene was only found present in five out of 252 assessed genomes. In this case, we observed a perfect gene–trait correlation between the presence of the cluster and their ability to utilize FHMO, in contrast with the situation observed with the genes involved in the LNT degradation.

Overall, this study provides an insight into the HMO catabolism in the *B. pseudocatenulatum* species, expanding our knowledge regarding fucosylated substrates able to be utilized by some *B. pseudocatenulatum* novel strains as well as the identification and characterization of the genes involved in LNT degradation. These results provide an insight into the potentially key role that certain strains of this taxon may play in the development of the infant gut, despite not being the most abundant species found in infants.

## MATERIALS AND METHODS

### Bacterial strains, plasmids, and culture conditions

*B. pseudocatenulatum* strains were routinely cultivated under anerobic conditions at 37°C in modified de Man Rogosa Sharpe (mMRS) medium prepared from first principles and supplemented with 0.5% lactose and 0.06% cysteine-HCl (24).

*B. pseudocatenulatum* cultures were cultivated under anerobic conditions in a modular atmosphere-controlled system (Davidson and Hardy, Belfast, Ireland) at 37°C. *Lactococcus cremoris* strains were cultivated in M17 broth (Oxoid Ltd., Basingstoke, England) containing 0.5% (wt/vol) glucose at 30°C.

### Growth assessment

Growth profiles of *B. pseudocatenulatum* strains were determined manually in mMRS Rogosa using 0.5% (wt/vol) of 2FL, 3FL, LNT, or LNnT as a sole carbon source. Five milliliters of media supplemented with 0.5% (wt/vol) of the HMO and 0.06% (wt/vol) of cysteine-HCl was inoculated with 100 μL of an overnight culture normalized to an OD_600nm_ of 2. A tube containing media with the corresponding sugar was used in each experiment as the blank. All cultures were cultivated under anerobic conditions for a duration of 24 hours. The anerobic chamber uses a standard anerobic mix of 10% hydrogen, 10% carbon dioxide, and 80% nitrogen.

Growth performance of the strains in the FHMOs (2’FL, 3FL, DFL, LNFPI, LNFPII, and LNPIII) was determined using a microplate spectrophotometer due to the limited availability of these substrates.

### Nucleotide sequence analysis

Sequence data were obtained from Artemis-mediated genome annotations of *B. pseudocatenulatum* strains and *B. kashiwanohense* APCKJ1 (14). Database searches were performed using non-redundant sequences accessible at the National Centre for Biotechnology Information (http://www.ncbi.nlm.nih.gov) using BLAST. Sequences were verified and analyzed using the Benchling (USA).

### DNA manipulations

Chromosomal DNA was isolated from *B. pseudocatenulatum* MM0196 using 1.5 mL of an overnight culture using the Genelute bacterial genomic DNA kit (Sigma). Plasmid DNA was isolated from *L. cremoris* using the GeneJET Plasmid Miniprep Kit (Thermo Scientific, UK). All restriction enzymes and T4 DNA ligase were used according to the supplier’s instructions (New England Biolabs, UK). Synthetic single-stranded oligonucleotide primers used, specified in Table 2, were synthesized by Eurofins (Ebersberg, Germany). Standard PCRs were performed using Dreamtaq (Thermo Scientific, USA). PCR products were visualized by Greensafe following agarose gel electrophoresis (1% agarose). PCR fragments were purified using the Roche High Pure PCR purification kit (Roche Diagnostics, Basel, Switzerland). Plasmid DNA was transformed into *L. cremoris* NZ9000 by electroporation according to published protocols.

**TABLE 2:** Primers used in this study^*a*^

Primer name	Sequence (5’−3’)	Description
0505_PstI_Fw	TGCATC**CTGCAG**ATGCATCACCATCACCATCACCATCACCATCACACTCAACGTAGAGCCTATCG	To amplify the gene 0505
0505_HindIII_Rv	AACGAT **AAGCTT**TTACTTCTTGGTGACG	To amplify the gene 0505
1726_XbaI_Fw	TGCATC**TCTAGA**ATGCATCACCATCACCATCACCATCACCATCACGCAAGCGAGAACACCAGCAAAT	To amplify the gene 1726
1726_HindIII_Rv	AACGAT **AAGCTT**TTATTTGCTGATTCGTCGAAGC	To amplify the gene 1726
1809_XbaI_Fw	TGCATC**TCTAGA**ATGCATCACCATCACCATCACCATCACCATCACGCAAACAGCAATCGTG	To amplify the gene 1809
1809_HindIII_Rv	AACGAT**AAGCTT**TTACTTCTTGGTGACGATCACG	To amplify the gene 1809

^*a*^ Restriction sites incorporated into oligonucleotide primer sequences are indicated in bold, and histidine tags incorporated into nucleotide primer sequences are indicated in italics.

### RNA-seq analysis

The strain MM0196 was grown in lactose and LNT as a sole carbon source (0.5%) in new modified Rogosa media supplemented with 0.06 cysteine until they reached an OD_600nm_ of 0.5. Five milliliters of the culture was centrifuged at 5,500 rpm at 4°C and immediately resuspended in 500 μL of Zymo RNA shield Solution 2X and stored at −80°C. Triplicates of the samples were prepared. The cell pellets were sent in dry ice to BaseClear (Leiden, Belgium) that performed the RNA extraction using the ZymoBIOMICS RNA Miniprep Kit (Zymo R2001) extraction kit and RNA sequencing using NovaSeq 6000 (Illumina).

### Construction of overexpression vectors, protein overproduction, and purification

For the construction of recombinant plasmids, various DNA fragments containing the targeted genes were amplified with Q5 high-fidelity polymerase (New England Biolab, UK) using MM0196 chromosomal DNA extracted with the Genelute bacterial genomic DNA kit (Sigma) as a template and employing primer pairs as specified in Table 2. Ten histidine residues were incorporated at the N-terminal region of the protein in order to facilitate the purification, the sequence corresponding to this region was incorporated in the primers, after the ATC. In all cases, the amplicons include the sequence of the complete gene minus the ATC, which was incorporated in the primers. The amplicon LnTA, which includes the gene MM0196_0505 and plasmid pNZ8150, were digested with PstI and HindIII (New England Biolabs, UK). Amplicons NahA and LacZ, including the complete ORF MM0196_1726 and MM0196_1809 respectively; were digested along with the plasmid pNZ8150 with HindIII and XbaI (New England Biolabs, UK). All PCR products and their corresponding plasmids were ligated using T4 ligase (New England Biolabs, UK). The ligation mix was introduced by electroporation into *L. cremoris* NZ9000 and transformants were selected with 10 μg/mL chloramphenicol. Transformants were screened by plasmid extraction and digestion analysis as well as PCR using the primers described on Table 2. All constructs were verified by sequencing (commercially performed by SNPsaurus, US) Table 3.

**TABLE 3:** Plasmids and strains used for cloning in this study

Strain or plasmid	Description	Reference or source
*Lactococcus cremoris* NZ9000	Cloning host	(25)
Plasmids		
pNZ8150	Cm^r^, cloning vector carrying the nisin-inducible promoter	(26)
pNZ8150:0505	Cm^r^; containing *LntA*	This study
pNZ8150:1726	Cm^r^; containing *NahA*	This study
pNZ8150:1809	Cm^r^; containing LacZ	This study

About 800 mL of the recombinant strains was grown in M17 media supplemented with 0.5% glucose up to an OD_600nm_ of 0.4, induced with 2 mL of the filtered sterilized supernatant of a nisin producer strain, and grow at the same conditions for another 4 hours. Cells were harvested by centrifugation and protein purification achieved following the previously published protocol. The pellets were then stored at −20°C overnight. The His-tagged proteins were purified by affinity chromatography using a Ni-NTA column (Qiagen, UK).

Purified proteins were dialysed and concentrated using Amicon Ultra-4 4 mL–10 KDa by combining all elution aliquots and washing with 10 mL of 20 mM MOPS buffer adjusted to pH 7. The protein concentration was assessed using Qubit using the manufacturer’s instructions.

### Enzymatic reactions

Enzymatic reactions were prepared in a volume of 1 mL and using an initial concentration of 1 mg/mL of the substrate (lactose or LNT) and adding 10 μg of protein and incubated for 24 hours at 37°C. Reactions were terminated by heat treatment at 85°C for 15 minutes.

### HPAEC-PAD analysis

For HPAEC-PAD analysis, a Dionex (Sunnyvale, CA) ICS-6000 system was used. Carbohydrate fractions from the above-mentioned hydrolysis assays (25-µL aliquots) were separated on a CarboPac PA1 analytical-exchange column (dimensions, 250  mm by 4  mm) with a CarboPac PA1 guard column (dimensions, 50  mm by 4  mm) and a pulsed electrochemical detector (ED40) in the PAD mode (Dionex). Elution was performed at a constant flow rate of 0.063  mL/min at 30°C. The following linear gradient of potassium hydroxide was used with 10  mM KOH: from 0 to 8 minutes, 10  mM; from 8 to 18  minutes, 10–40 mM; from 18 to 5  minutes, 40 mM; from 25 to 30  minutes, 10 mM. Chromatographic profiles of standard carbohydrates were used for comparison of the results of their breakdown by LntA_ps-His_, NahA_ps-His,_ and LacZ9_ps-His_ proteins. Chromeleon software (version 7; Dionex Corporation) was used for the evaluation of the chromatograms obtained. A 1  mg/mL stock solution of each of the carbohydrates, as well as their putative breakdown products (where available) used as reference standards, was prepared by dissolving the sugar in Milli-Q water.

## Data Availability

The RNAseq raw data have been submitted to SRA and are available under BioProject PRJNA1120230.
